# Afatinib, an irreversible ErbB family blocker, with protracted temozolomide in recurrent glioblastoma: A case report

**DOI:** 10.18632/oncotarget.5297

**Published:** 2015-09-21

**Authors:** Jad Alshami, Marie-Christine Guiot, Scott Owen, Petr Kavan, Neil Gibson, Flavio Solca, Agnieszka Cseh, David A. Reardon, Thierry Muanza

**Affiliations:** ^1^ Clinical Research Unit, Montreal Neurological Institute and Hospital, McGill University Health Center, Montreal, Canada; ^2^ Drug Metabolism & Pharmacokinetics, Boehringer Ingelheim Pharma GmbH & Co. KG, Biberach, Germany; ^3^ Boehringer Ingelheim RCV GmbH & Co. KG, Vienna, Austria; ^4^ Center for Neuro-Oncology, Dana-Farber Cancer Institute, Boston, Massachusetts, USA; ^5^ Radiation Oncology, Jewish General Hospital, Montreal, Canada

**Keywords:** afatinib, temozolomide, glioblastoma, next-generation sequencing, epidermal growth factor receptor

## Abstract

There are few effective treatments for recurrent glioblastoma multiforme (GBM). We present a patient with recurrent GBM who achieved a prolonged response to treatment with afatinib, an irreversible ErbB family blocker, plus temozolomide. A 58-year-old female patient was diagnosed with multifocal primary GBM. After surgical resection, first-line therapy comprised radiotherapy and temozolomide. Following disease progression after 3 temozolomide cycles, the patient entered a phase I/II clinical trial of afatinib (20–40 mg daily for 28 days) plus temozolomide (50 mg/m^2^ every 21/28 days). Next-generation sequencing analysis of the brain tumor specimen was performed. At the last assessment, 63 treatment cycles had been completed and the patient had survived for ~5 years since recurrence. Significant disease regression was observed after 5 cycles and was maintained during long-term follow-up. Adverse events were consistent with the known tolerability profile of afatinib and were managed by treatment interruption/dose reduction. The patient had several epidermal growth factor receptor *(EGFR)* aberrations, including gene amplification and *EGFRvIII* positivity. Three somatic mutations were identified, including an unprecedented extracellular-domain substitution (D247Y). The patient has survived ~6-fold longer than normally expected in patients with recurrent GBM. The complex *EGFR* genotype may underlie sustained response to afatinib plus temozolomide.

## INTRODUCTION

Glioblastoma multiforme (GBM) is the most frequent, highly malignant primary tumor of the central nervous system [1], accounting for approximately 12–15% of all brain tumors [2]. The current standard of care is temozolomide plus radiotherapy [3]. However, almost all GBMs recur after first-line therapy and few second-line options have been identified that provide sustained clinical benefit [4]. Consequently, most patients die soon after tumor recurrence; median overall survival after disease progression is approximately 6–9 months [4, 5]. However, a small proportion of patients can survive for considerably longer [6].

As GBM is highly heterogeneous, optimal treatment tailored to the individual is difficult [5]. Nevertheless, several potential prognostic factors of survival have been identified and include the patient's age, and performance status, tumor location, and extent of surgical resection [7]. Furthermore, developments in genomic technology have facilitated the identification of molecular markers that could potentially drive treatment decisions. For example, methylation status of the gene encoding O^6^-methylguanine-DNA methyltransferase (MGMT) predicts response to temozolomide-based regimens in elderly patients [8]. Molecular studies have also identified markers that provide insights into the pathogenesis of GBM and potentially identify rationale for drug targets. For example, heritable genetic aberrations of the ErbB family of receptors, particularly the epidermal growth factor receptor (EGFR), has been implicated in GBM progression [9]. Overexpression of *EGFR* has been identified in 50–60% of GBM cases and is generally thought to confer poor prognosis [10–12], particularly in the presence of the *EGFRvIII* mutation [13].

To date, clinical trials with targeted agents in patients with GBM have been disappointing. For example, bevacizumab and cilengitide have been investigated in a first-line treatment setting, but these studies failed to show improvements in survival [14–16]. Furthermore, despite the likely role of EGFR in the pathogenesis of disease, reversible EGFR tyrosine kinase inhibitors (gefitinib and erlotinib) do not appear to be effective for recurrent GBM [17–19]. Recently, a phase I/II study assessed afatinib, a potent irreversible ErbB family blocker, with or without protracted temozolomide, in patients with recurrent GBM [20]. The rationale for this study was the observation that afatinib inhibits proliferation of cells with *EGFR* mutations that are commonly found in GBM, including *EGFRvIII* and *R108K* [21, 22]. Furthermore, unlike erlotinib and gefitinib, cytochrome P450 metabolism of afatinib is negligible [23], thus facilitating combination with chemotherapy or some anti-epileptic drugs. Also, clinical studies have shown afatinib to be effective in several tumor types, notably non-small cell lung cancer including patients with acquired resistance to gefitinib or erlotinib [24–28]. Unfortunately, the phase I/II study demonstrated that afatinib monotherapy, and afatinib plus temozolomide, had limited activity in unselected patients with recurrent GBM. However, certain selected patient populations (including patients with high levels of EGFR vIII immunoreactivity, *EGFR* amplification, or *PTEN* loss) appeared to have promising response and durable progression-free survival.

Here, we present a patient with multifocal recurrent GBM who demonstrated a remarkable response to treatment with afatinib plus temozolomide. We undertook broad molecular analysis on this patient's tumor to assess possible mechanistic explanations for the sustained clinical benefit that was observed. Informed consent has been obtained.

## CASE PRESENTATION

This right-handed, 58-year-old, previously healthy female patient presented in October 2009 with constant right frontal headache, mild weakness of the left side, gait disturbance, and behavioral changes (anger, forgetfulness).

### Radiological analysis

**Figure 1:**
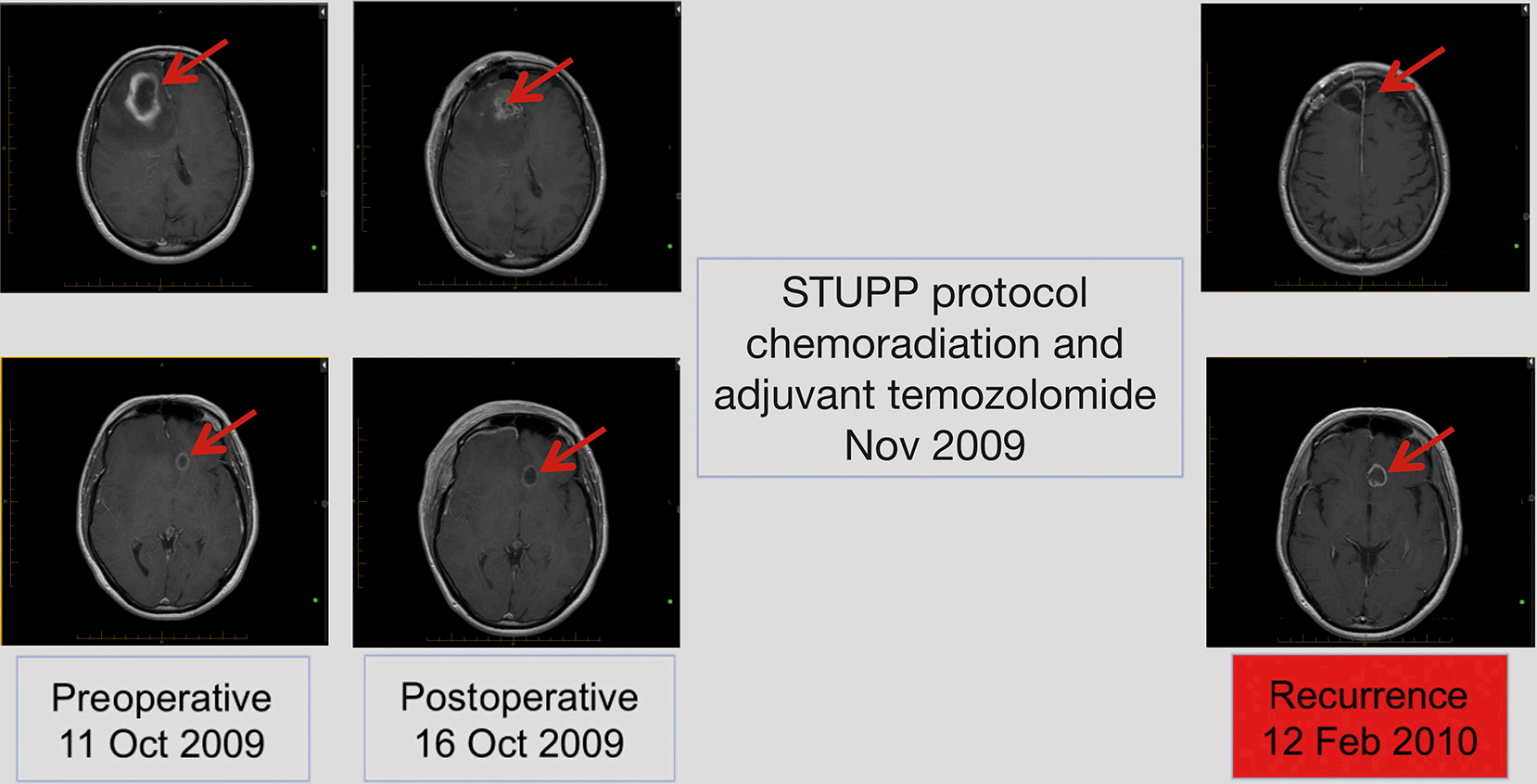
Radiological disease assessments relating to first-line treatment (surgical resection and STUPP protocol)

Three lesions were identified from radiological assessment (Figure 1). These included a right frontal lesion (4.0 × 3.7 × 4.9 cm) with surrounding edema and mass effect on the adjacent right frontal horn (9 mm to the left of midline); a subcortical left basal frontal area lesion (maximum diameter 1.3 cm) with thick marginal enhancement and central cystic/necrotic appearance, and a left inferior frontal gyrus lesion evident in T2 and Flair images.

### Surgical resection

Subtotal resection of the right frontal lesion was performed in October 2009. Postoperative reduction in tumor size was evident upon radiological assessment (Figure 1). The patient was diagnosed with multifocal glioma and pathological findings were consistent with GBM World Health Organization (WHO) grade IV. Tumor MGMT promoter and isocitrate dehydrogenase 1 (IDH1) status were determined to be methylated and wild-type, respectively. Two weeks following surgery, the patient developed a deep vein thrombosis (DVT) in the right lower extremity and a pulmonary embolism. Consequently, the patient was treated with heparin followed by low molecular weight heparin. Within 10 days, she had fully recovered from the DVT.

### Treatments administered

First-line treatment, initiated in November 2009, comprised the STUPP protocol: radiotherapy (60 Gy over 6 weeks; intensity modulated radiotherapy [IMRT]) with concurrent oral temozolomide (75 mg/m^2^ daily for 42 days), followed by adjuvant temozolomide 150–200 mg/m^2^ every 5/28 days [29]. The patient tolerated the concurrent treatment well. However, in February 2010, disease progression was observed by magnetic resonance imaging (MRI) after 3 cycles of adjuvant temozolomide (increase in left frontal lesion with mass effect; Figure 1).

**Figure 2:**
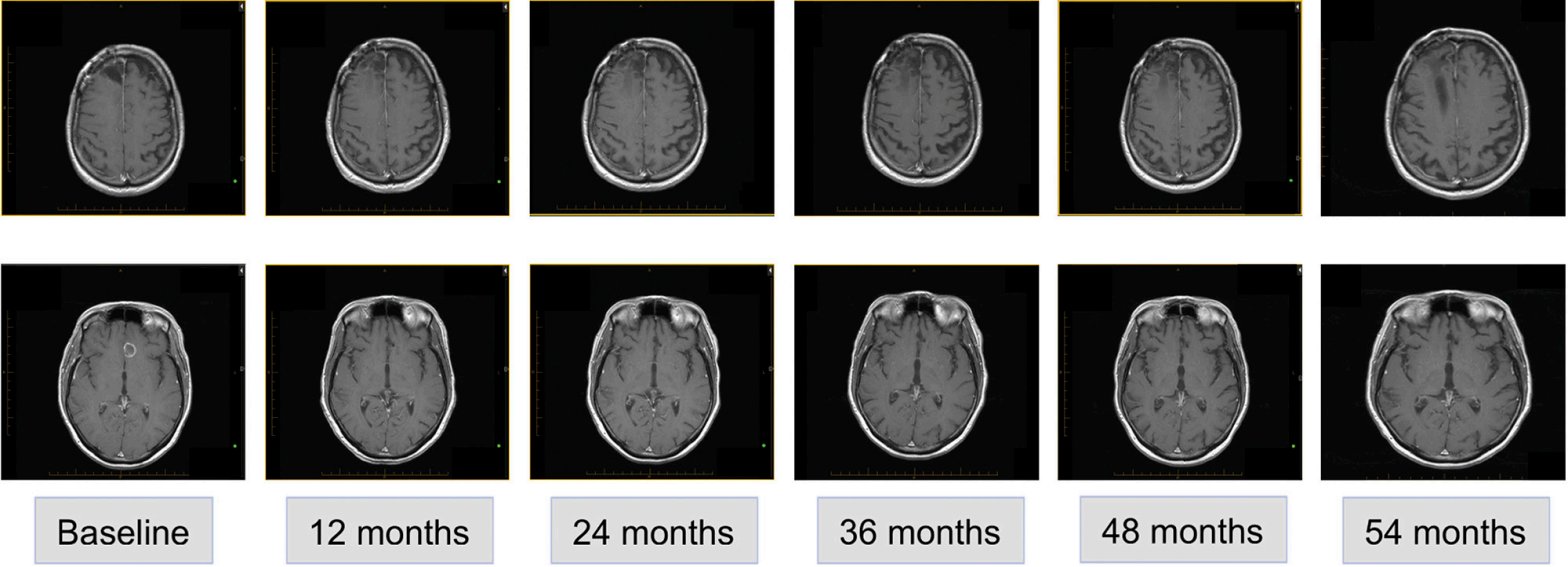
Radiological disease assessments relating to second-line treatment (afatinib and temozolomide)

Second-line treatment, initiated in April 2010, was determined according to participation in a clinical trial of afatinib with or without daily temozolomide. The patient was randomized to the combined afatinib (20–40 mg daily for 28 days) and temozolomide (50 mg/m^2^ orally [p.o.] every 21/28 days) arm. After 1 cycle of second-line treatment, MRI revealed minimal decrease in lesion size. After 5 cycles, significant disease regression was observed and maintained in subsequent assessments to 54 months (Figure 2). A full spine MRI performed in November 2014 showed no evidence of metastases in the cervical, thoracic or lumbar regions and the most recent MRI (January 2015) showed stable disease. At the time of writing (April 2015), the patient had completed 63 cycles and treatment was ongoing.

### Adverse events

In total, the patient experienced four incidences of grade III adverse events that were considered to be at least probably related to second-line treatment: one incidence of maculopapular rash and three incidences of paronychia. The maculopapular rash occurred during the third cycle and was managed with a 2-week interruption of treatment followed by a dose reduction (afatinib from 40 to 30 mg p.o. daily; temozolomide from 140 to 90 mg p.o. daily). Grade III paronychia occurred during cycle 16, cycle 20 (leading to a dose reduction of afatinib from 30 to 20 mg p.o. daily), and cycle 36 (leading to a treatment interruption of 7 weeks). Dose reductions led to marked improvement in symptoms.

Other drug-related adverse events included grade I nausea and vomiting (cycle 1; managed with anti-emetics), grade II diarrhea (cycle 1; managed with loperamide), grade I rash (cycles 1 and 2; managed with hydrocortisone cream), weight loss, and fatigue.

The patient's neurologic and physical status were stable (Karnofsky Performance Status > 90) throughout second-line treatment.

### Molecular analysis

DNA was isolated from a brain tumor specimen from the primary resection and subjected to FoundationOne™ next-generation sequencing [30]. The entire coding sequence of 236 cancer-related genes, plus 47 introns from 19 genes often rearranged or altered in cancer, was assessed. Fifteen cancer-related gene alterations were evident.

A number of *EGFR* aberrations were observed (Table 1). The patient was positive for *EGFR* amplification (estimated copy number of 60) and was likely positive for the *EGFRvIII* mutation (the extent of the *EGFRvIII* variant is difficult to quantify with next-generation sequencing). It is not unusual to observe amplification of both wild-type *EGFR* and *EGFRvIII* in the same tumor; *EGFRvIII* is rarely observed in isolation [31]. Two subclonal somatic mutations were observed (2% of reads), P596L and G598V. Both of these mutations have previously been identified in patients with GBM [21]. Interestingly, an additional unprecedented variant of unknown significance was observed in the extracellular domain of EGFR (D247Y; 89% of reads). This could potentially be a rare single nucleotide polymorphism (SNP), but could also be a somatically acquired allele that may possibly be linked to the observed clinical response to afatinib.

**Table 1:** Details of gene alterations detected in 15 genes

Number	Gene	Description
1	***EGFR***	Amplification of full gene, estimated gene copy number = 60
2	***EGFR***	Known somatic mutation P596L (c.1787C > T)
3	***EGFR***	Known somatic mutation G598V (c.1793G > T)
4	***EGFR***	Known somatic mutation *EGFRvIII*
5	***EGFR***	Variant of unknown significance D247Y (c.739G > T)
6	***PTEN***	Known somatic mutation R130* (c.388C > T)
7	***CDKN2A (p16)***	Homozygous deletion of full gene
8	***CDKN2B (p15)***	Homozygous deletion of full gene
9	***BAP1***	Variant of unknown significance V447I (c.1339G > A)
10	***BCORL1***	Variant of unknown significance T1111M (c.3332C > T)
11	***C17orf39***	Variant of unknown significance N285Y (c.853A > T)
12	***CDH1***	Variant of unknown significance P30T (c.88C > A)
13	***EPHA5***	Variant of unknown significance M987T (c.2960T > C)
14	***ESR1***	Variant of unknown significance H6Y (c.16C > T)
15	***GRIN2A***	Variant of unknown significance C800 (c.2400C > A)
16	***MAP3K1***	Variant of unknown significance S939C (c.2816C > G)
17	***NOTCH3***	Variant of unknown significance R1669H (c.5006G > A)
18	***STAG2***	Variant of unknown significance splice (c.2026–1G > C)
19	***IKZF1***	Amplification of full gene, estimated gene copy number = 51

A: adenine; C: cytosine; EGFR: epidermal growth factor receptor; G: guanine; GBM: glioblastoma; SNP: single nucleotide polymorphism; T: thymine.

Other genetic aberrations of interest included a null mutation in *PTEN* and deletion of *CDKN2A* and *B*, all of which are common genetic features of GBM. A number of other genes were found to have single base-pair changes, including *BAP1*, *BCORL1*, *C170rf39*, *CDH1*, *EPHA5*, *ESR1*, *GRIN2A*, and *MAP3K1*. It is likely that all of these aberrations represent rare heterozygous SNPs without any functional relevance (Table 1). Of note, several other common molecular genetic features of GBM, such as mutations in *TP53*, *NF1*, and *IDH1*, were absent in this patient. In independent analysis, the *MGMT* promoter was found to be methylated.

## AFATINIB IN MOUSE XENOGRAFT MODELS OF GBM

**Figure 3:**
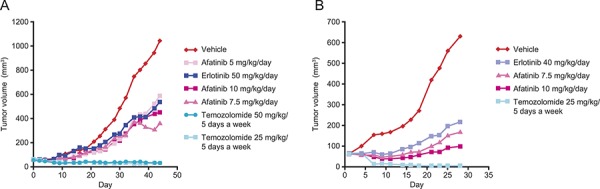
Effect of afatinib, erlotinib and temozolomide in two different human patient-derived xenograft models of glioblastoma **A.** GB218 tumor growth kinetics. Groups of GB218 tumor-bearing mice (*n* = 7/group) were treated orally in weekly schedules either with afatinib 10, 7.5 or 5 mg/kg/day, erlotinib 50 mg/kg/day, temozolomide 25 or 50 mg/kg/5 days, or with the vehicle only. Median tumor volumes are plotted over time. Day 1 was the first and Day 44 the final day of treatment. **B.** GB138 tumor growth kinetics. Groups of GB138 tumor-bearing mice (*n* = 7/group) were treated orally in weekly schedules either with afatinib 10 or 7.5 mg/kg/day, erlotinib 40 mg/kg/day, temozolomide 25 mg/kg/5 days, or with the vehicle only. Median tumor volumes are plotted over time. Day 1 was the first and Day 28 the final day of treatment.

The anti-tumor activity of afatinib has been evaluated in two different glioblastoma models derived from adult patients. These experiments were performed before trial initiation with the aim of illustrating that afatinib could have single-agent activity in glioblastoma models. Of note, both models have some degree of genetic similarity with the tumor profile of the patient described in this report (likely positive for *EGFRvIII mutation* and positive for EGFR amplification). The GB218 glioblastoma model is characterized by the presence of *EGFRvIII* mutation. In this model, monotherapy treatment (44 days) with afatinib (10, 7.5 or 5mg/kg/day) or erlotinib (50mg/kg/day) resulted in tumor growth inhibition (TGI) of 60.0, 69.3, 46.9, and 52.2%, respectively (Figure 3A). These doses are below the usual maximum tolerated doses in mice. GB218 also displayed sensitivity to temozolomide (50 or 25 mg/kg/day) resulting in complete suppression of tumor growth (TGI = 103.4%). The GB138 glioblastoma model is characterized by *EGFR* gene copy number gain (EGFR amplification). In this model, monotherapy treatment (28 days) with afatinib (10 or 7.5 mg/kg/day) or erlotinib (40mg/kg/day) resulted in TGI of 91.5, 81.8 and 72.7%, respectively (Figure 3B). Temozolomide (25 mg/kg/5 days on - 2 days off) resulted in complete suppression of tumor growth (TGI = 110.5%).

## DISCUSSION

This case report describes a sustained clinical and radiographic response in a patient with recurrent multifocal GBM. Since progression on first-line therapy, the patient has survived for 60 months, approximately 6 times longer than the median overall survival generally observed in patients with recurrent GBM. It is not possible to say with certainty that these striking observations are solely attributable to treatment with afatinib and temozolomide. Moreover, pseudo-progression cannot be definitively excluded because the tumor is *MGMT*-methylated and disease progression was observed only 4 months after chemoradiation treatment.

This patient was participating in a clinical trial of afatinib with or without temozolomide for recurrent GBM (NCT00727506) [20]. The main finding of this study was that afatinib has limited effect on survival in unselected patients. Overall, median progression-free survival in patients treated with afatinib plus temozolomide (*n* = 39) was 1.5 months versus 1.9 months in patients treated with temozolomide only (*n* = 39). Nevertheless, subanalysis suggested that patients with certain molecular characteristics may benefit from combination therapy versus temozolomide monotherapy. For example, median progression-free survival was 2.7 versus 1.0 months in patients with *EGFR* amplification, and 2.7 versus 1.9 months in patients with *PTEN* loss. The present patient exhibited characteristics associated with a stronger afatinib response: *EGFR* amplification, *EGFRvIII* positivity, and a null mutation in *PTEN*. Our preclinical data in mouse xenograft models demonstrate that tumors with these EGFR aberrations are sensitive to tyrosine kinase inhibitors.

We have undertaken broad molecular analysis of the patient to try to more clearly define the molecular aberrations that could potentially identify patients who might achieve significant clinical benefit from afatinib plus temozolomide. The patient's overall molecular pathology was largely consistent with the ‘classical GBM’ subtype proposed by Verhaak, et al. [32], which is characterized by *EGFR* amplification and a lack of abnormalities in *TP53*, *NF1*, *PDGFRα*, and *IDH1*. It is possible that EGFR amplification and *EGFRvIII* positivity could underlie the encouraging response to afatinib plus temozolomide. However, 3 additional *EGFR* aberrations were also identified. Two of these aberrations, P596L and G598V, were clonally rare within the tumor and are therefore unlikely candidates for driving response to combination therapy. However, the third aberration, D247Y, appeared to be clonally amplified in the tumor and could therefore conceivably contribute to the observed response to afatinib plus temozolomide. Interestingly, this aberration affects the extracellular domain of EGFR. A previous study has shown that variants in the extracellular domain are relatively common in patients with GBM (13.6%) and confer sensitivity to EGFR tyrosine kinase inhibitors [21]. Based on these observations, we hypothesize that the complex *EGFR* genotype comprising extracellular aberrations in concert with focal amplification and the *EGFRvIII* mutation may underlie the observed sensitivity to afatinib and temozolomide. This hypothesis may be tested further in a suitable *in vitro* model. Moreover, it would be interesting to assess patients who have been treated with the combination in the phase I/II trial for *EGFR* extracellular aberrations. It is important to note that the tumor specimen sequenced was from initial resection, prior to any treatment; as such, the profile may have evolved upon exposure to subsequent therapies, but we cannot speculate on the nature of any treatment-driven evolution.

Adverse events in this case were manageable and in line with previous studies of the safety profiles of afatinib and temozolomide, including the GBM trial [20, 24, 26, 33]. In relation to safety, the GBM study also reported a lack of pharmacokinetic interactions between afatinib and temozolomide [20], supporting a strategy of co-administration of these drugs.

In conclusion, we describe a novel extracellular *EGFR* mutation in a recurrent GBM patient who demonstrated uncharacteristically prolonged survival following afatinib plus daily temozolomide. Studies to confirm the criteria for predicting afatinib response, and to determine the optimal dosing regimen, are awaited with interest.

### Ethics statement

Investigation has been conducted in accordance with the ethical standards and according to the Declaration of Helsinki and according to national and international guidelines and has been approved by the authors’ institutional review board.
